# HBx sensitizes hepatocellular carcinoma cells to lapatinib by up-regulating ErbB3

**DOI:** 10.18632/oncotarget.6337

**Published:** 2015-11-16

**Authors:** Jhen-Yu Chen, Yun-Ju Chen, Chia-Jui Yen, Wen-Shu Chen, Wei-Chien Huang

**Affiliations:** ^1^ The Ph.D. Program for Cancer Biology and Drug Discovery, China Medical University and Academia Sinica, Taichung, Taiwan; ^2^ Graduate Institute of Cancer Biology, China Medical University, Taichung, Taiwan; ^3^ Department of Biological Science & Technology, I-Shou University, Kaohsiung, Taiwan; ^4^ School of Medicine for International Students, I-Shou University, Kaohsiung, Taiwan; ^5^ Department of Medical Research, E-Da Hospital, Kaohsiung, Taiwan; ^6^ Internal Medicine, National Cheng-Kung University, Tainan, Taiwan; ^7^ Center for Molecular Medicine, China Medical University and Hospital, Taichung, Taiwan; ^8^ Department of Biotechnology, Asia University, Taichung, Taiwan

**Keywords:** HBx, hepatocellular carcinoma, EGFR family, lapatinib, target therapy

## Abstract

Poor prognosis of hepatitis B virus (HBV)-associated hepatocellular carcinoma (HCC) involves HBV X protein (HBx)-induced tumor progression. HBx also contributes to chemo-resistance via inducing the expressions of anti-apoptosis and multiple drug resistance genes. However, the impact of HBx expression on the therapeutic efficacy of various receptor tyrosine kinase inhibitors remains unknown. In this study, our data showed that HBx overexpression did not alter the cellular sensitivity of HCC cell lines to sorafenib but unexpectedly enhanced the cell death induced by EGFR family inhibitors, including gefitinib, erlotinib, and lapatinib due to ErbB3 up-regulation. Mechanistically, HBx transcriptionally up-regulates ErbB3 expression in a NF-κB dependent manner. In addition, HBx also physically interacts with ErbB2 and ErbB3 proteins and enhances the formation of ErbB2/ErbB3 heterodimeric complex. The cell viability of HBx-overexpressing cells was decreased by silencing ErbB3 expression, further revealing the pivotal role of ErbB3 in HBx-mediated cell survival. Our data suggest that HBx shifts the oncogenic addiction of HCC cells to ErbB2/ErbB3 signaling pathway via inducing ErbB3 expression and thereby enhances their sensitivity to EGFR/ErbB2 inhibitors.

## INTRODUCTION

Hepatocellular carcinoma, the most malignant type of liver cancers, is the third leading cancer–related mortality [1]. Hepatitis B virus (HBV) and Hepatitis C virus (HCV) infections cause cirrhosis and subsequently contribute to the tumorigenesis in most of HCC patients [2]. These risk factors inactivate p53 transcriptional functions, induce inflammation response, and produce oxidative stress to promote HCC development by increasing cell proliferation, loss of growth, and genetic alternation [3, 4]. HBV, the first virus found to associate with human cancer, contributes to HCC development in Asia and South Africa. HBV-associated HCC is characterized by highly chemotherapy resistance [5, 6]. HBV X protein (HBx) is encoded in the smallest open reading frame X, and has been well demonstrated to play a critical role in HBV-induced HCC development. HBx also mediates chemo-resistance in HCC by modulating a number of cellular genes involved in NF-κB pathway, multiple-drug resistance, cell cycle progression, apoptosis, and genetic stability [7–10]. However, the impact of HBx expression on the therapeutic efficacy of targeted therapy against to receptor tyrosine kinase (RTK) remains unknown.

Recent understanding of the complex signaling network involved in HCC proliferation, progression and survival leads to the development of sorafenib to this disease [11, 12]. Sorafenib targets several key signal transduction pathways, oncogenes, growth factors and their receptors, including vascular endothelial growth factor (VEGF)/VEGF receptor (VEGFR), platelet-derived growth factor (PDGF)/PDGF receptor (PDGFR), and their downstream Raf kinase. Activation or expression of these molecules has been implicated in resistance to apoptosis, cell proliferation, stimulation of angiogenesis, invasiveness and metastasis of HCC [13–15] and is also correlated with its poor prognosis [16, 17]. In addition to inducing cell death and inhibiting angiogenesis through targeting VEGFR and PDGFR in a Raf-dependent manner [18], sorafenib also suppresses tumor growth by down-regulation of Mcl-1 and enhancement of ER stress in a Raf-independent manner [19]. However, loss of therapeutic efficacy occurs ultimately despite the initial responses to sorafenib [20–22]. Activation of JAK/STAT, NF-κB, and PI3K/Akt pathway [23–25], overexpression of HIF-1α and EGFR [26, 27], and induction of EMT and autophagy [28, 29] have been proposed to confer the acquired resistance to sorafenib.

In addition to VEGFR, overexpression of ErbB family including EGFR, ErbB2, and ErbB3 also associate with HCC formation [30]. EGFR is activated by binding of its specific ligands and stimulates its downstream PI3K/Akt and Ras/Raf/MEK/MAPK signaling pathway activation, leading to cell proliferation, cell survival, metastasis, and angiogenesis [31, 32]. Therefore, its overexpression is a negative prognostic factor for HCC [33]. Targeting EGFR tyrosine kinase activity by pharmacological inhibitors such as erlotinib and gefitinib are approved for lung cancer. Lapatinib, an EGFR/ErbB2 dual inhibitor, is used for advanced ErbB2-positive breast cancer cells [34–36]. However, the response rate of HCC patients to these EGFR tyrosine kinase inhibitors (TKIs) is low, and combination of sorafenib with EGFR TKIs did not provide additional benefit to HCC patients [37, 38].

Previous studies have shown that HBx activates Ras/Raf/MEK/MAPK signaling pathway by elevation of VEGFR3 to antagonize pro-apoptosis [39]. HBx also has been reported to increase VEGF expression through up-regulation of mTOR pathway [40]. Regulations of EGFR and ErbB2 expression by HBx in HCC cell lines were also described in our previous reports [41, 42]. However, it is unclear whether these regulatory effects of HBx on VEGFR, EGFR family, and their downstream signaling further alter the anti-cancer activity of RTK TKIs. In this study, we addressed the impact of HBx on the cellular sensitivity of HCC cells to sorafenib and EGFR inhibitors, including gefitinib, erlotinib, and lapatinib. Our data showed that HBx overexpression did not alter the cellular sensitivity of HCC cell lines to sorafenib, but unexpectedly enhanced the cell death induced by EGFR family inhibitors through shifting the oncogenic addiction to ErbB2/ErbB3 signaling pathway via enhancing ErbB3 expression. These findings also suggest HBx and ErbB3 as the potential biomarkers for predicting the therapeutic efficacy of EGFR/ErbB2inhibitors in HCC patients.

## RESULTS

### Overexpression of HBx increases the sensitivity of HCC cell lines to EGFR/ErbB2 TKIs

**Figure 1:**
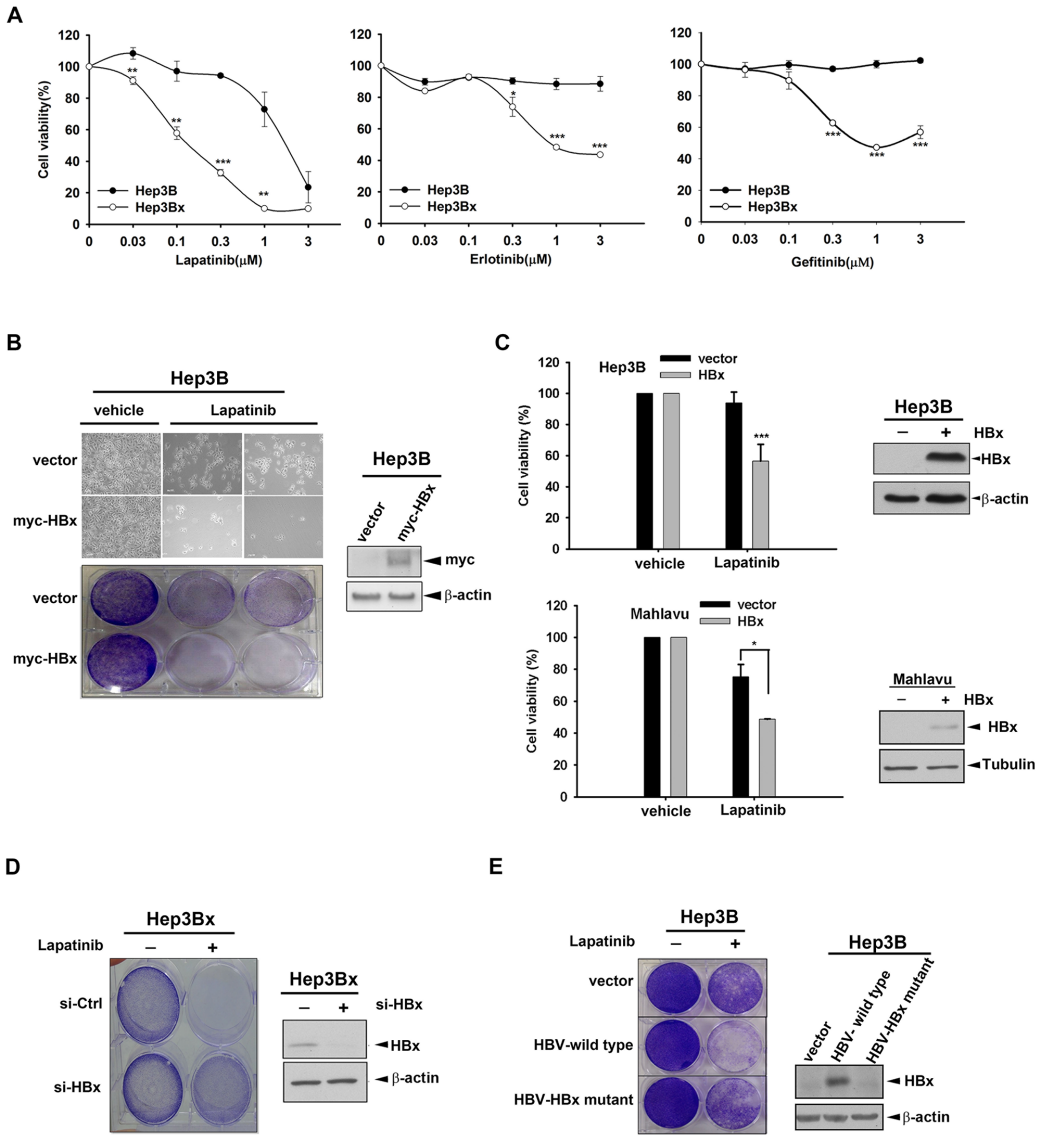
HBx overexpression sensitizes HCC cell lines to EGFR/ErbB2 TKIs **A.** The cell viability of Hep3B and Hep3Bx were determined by MTT assay after treatment with lapatinib, erlotinib, and gefitinib for 3 days. **B.** Hep3B cells were transiently transfected with myc-HBx followed by treatment with 1 μM lapatinib for 3 days. The cell viability was determined by crystal violet staining. **C.** Hep3B and Mahlavu cells were infected by lentivirus and treatment with 0.5 μM lapatinib for 3 days. The cell viability was determined by MTT assay. The protein expression of pDEST-HBx and β-actin was determined by Western blot. β-actin acts as an internal control. **D.** Hep3Bx cells were transfected with control and HBx siRNA for 3 days followed by treatment with 1 μM lapatinib. The cell viability was determined by crystal violet staining. The protein expression of HBx and β-actin was determined by Western blot. **E.** The control vector, HBV whole genome, and HBx-deleted HBV mutant were transiently transfected into Hep3B cells. After treatment with 1 μM lapatinib, the cell viability was detected by crystal violet staining. The protein expression of HBx and β-actin was determined by Western blot. Statistical analysis was performed by Student's *t* test. *, *p* < 0.05; **, *p* < 0.01; ***, *p* < 0.001 as compared to control group.

It has been reported that HBx may cause chemo-resistance in HCC cells [43]. Here, we investigated whether HBx also renders HCC cells more resistant to RTK TKIs. First, we compared the sensitivity of Hep3B HCC cell line and its HBx-overexpressing stable clone (Hep3Bx) to sorafenib, the standard TKI therapy for advanced HCC in clinic, by measuring the cell viability in MTT assays after treatment for 3 days. However, the inhibition of viability by sorafenib in these two cell lines had no significant difference (Supplementary Figure S1A), suggesting that HBx overexpression may not affect the anti-cancer activity of sorafenib in HCC cells. EGFR overexpression is commonly observed in HCC [30]. By examining the protein levels of EGFR family members in Western blot analysis, our data indicated that the expressions of EGFR, ErbB2, and ErbB3 were generally abundant in various HCC cell lines (Supplementary Figure S1B). Therefore, we then tested the impact of HBx expression on the cellular sensitivity of HCC cells to EGFR/ErbB2 TKIs. As shown in Figure 1A, the viability of Hep3B cells was decreased by EGFR/ErbB2 dual inhibitor lapatinib only at high concentration (3 μM), and was not affected completely by two EGFR-specific TKIs, erlotinib and gefitinib. Unexpectedly, HBx-overexpressing Hep3Bx cell line is much more sensitive to these drugs in comparison to its parental cell line. These results suggested that although ErbB family is commonly overexpressed in HCC cells, it might not be the major driver gene for tumor survival. Nevertheless, when HBx protein is overexpressed, the addicted pro-survival pathways in HCC cells may be modulated and switched to the ErbB family signaling, leading to the increase of sensitivity to EGFR/ErbB2 TKIs. Because lapatinib showed more obviously inhibitory effect on the viability of Hep3Bx cells than erlotinib and gefitinib did, we focus on lapatinib in the following experiments to address the underlying mechanisms.

To exclude the possibility that the increased sensitivity of HBx-overexpressing stable clone to lapatinib is due to the clonal effect, we transiently transfected myc-tagged HBx into Hep3B cells followed by treatment with lapatinib for 3 days in the clonogenic assay. The data showed that cell number of HBx-expressing cells was less than that of control cells in response to lapatinib treatment (Figure 1B, upper panel). After crystal violet staining, we can also observe higher sensitivity to lapatinib in HBx-expressing cells than in the control cells (Figure 1B, lower panel). Similar sensitization to lapatinib by lentivirally expressing HBx in Hep3B cells was obtained in MTT assay (Figure 1C, upper). We also tested whether HBx displays the same function in other HCC cell lines. Mahlavu and HepG2 cells were transiently transfected with HBx followed by lapatinib treatment in MTT assay. Similarly, HBx overexpression also increased lapatinib sensitivity in Mahlavu and HepG2 cells (Figure 1C, lower and Supplementary Figure S1C), suggesting a common lapatinib-sensitizing effect of HBx in HCC cells. To further confirm the essential role of HBx in sensitizing HCC cells to lapatinib, HBx was knocked down by siRNA in Hep3Bx cells in clonogenic assay and the data showed that silencing of HBx reduced the sensitization to lapatinib in Hep3Bx cells (Figure 1D). Furthermore, Hep3B cells transfected with HBV whole genome also showed higher sensitivity to lapatinib in clonogenic assays, but this effect was abolished when HBx was mutated (Figure 1E), supporting the enhancing effect of HBx on the anticancer activity of lapatinib in HCC cells.

**Figure 2:**
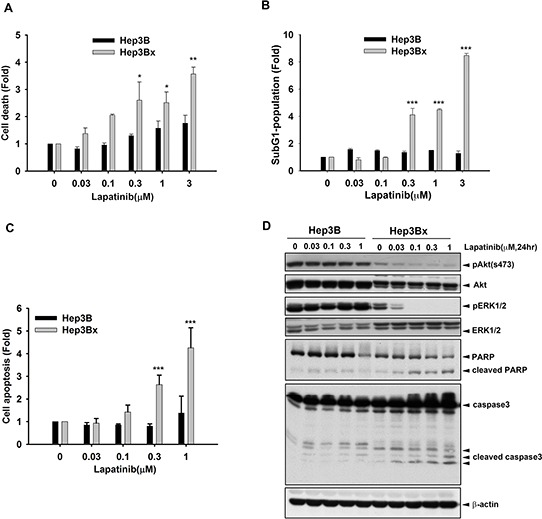
HBx enhanced the sensitization of Hep3B cells to lapatinib-induced apoptosis **A–C.** Hep3B and Hep3Bx were treated with indicated concentration of lapatinib for 5 days. The cells were trypsinized for cell number counting (A), PI staining for determining sub-G1 population (B), and PI and Annexin V double staining for determining apoptosis (C). **D.** Total lysate prepared from lapatinib-treated Hep3B and Hep3Bx cells were subjected to Western blot analysis with anti-pAkt(s473), anti-Akt, anti-pERK, anti-ERK, anti-PARP, anti-caspase3, and anti-β-actin antibodies. Statistical analysis was performed by Student's *t* test. *, *p* < 0.05; **, *p* < 0.01; ***, *p* < 0.001 as compared to control group.

We next investigated how HBx enhanced the anticancer activity of lapatinib in HCC cells. As shown in Figure 2A, the cell death induced by lapatinib was higher in Hep3Bx cells than their parental cells in cell counting assays. Consistently, the induction of sub-G1 population by lapatinib was much more in Hep3Bx cells than in Hep3B cells (Figure 2B). Hep3Bx cells treated with lapatinib followed by double staining with annexin V/PI in flow cytometry analysis showed more apoptotic population than Hep3B cells did (Figure 2C), indicating that HBx overexpression may enhance the pro-apoptotic effect of lapatinib. In supporting to this notion, treatment with lapatinib for 24 hours not only suppressed Akt and ERK activity but also induced more protein cleavage of PARP and Caspase3 in Hep3Bx cells than in Hep3B cells (Figure 2D). Taken together, these data showed that HBx sensitized HCC cell lines to lapatinib-induced apoptosis.

### HBx increases the protein and mRNA levels of ErbB3

**Figure 3:**
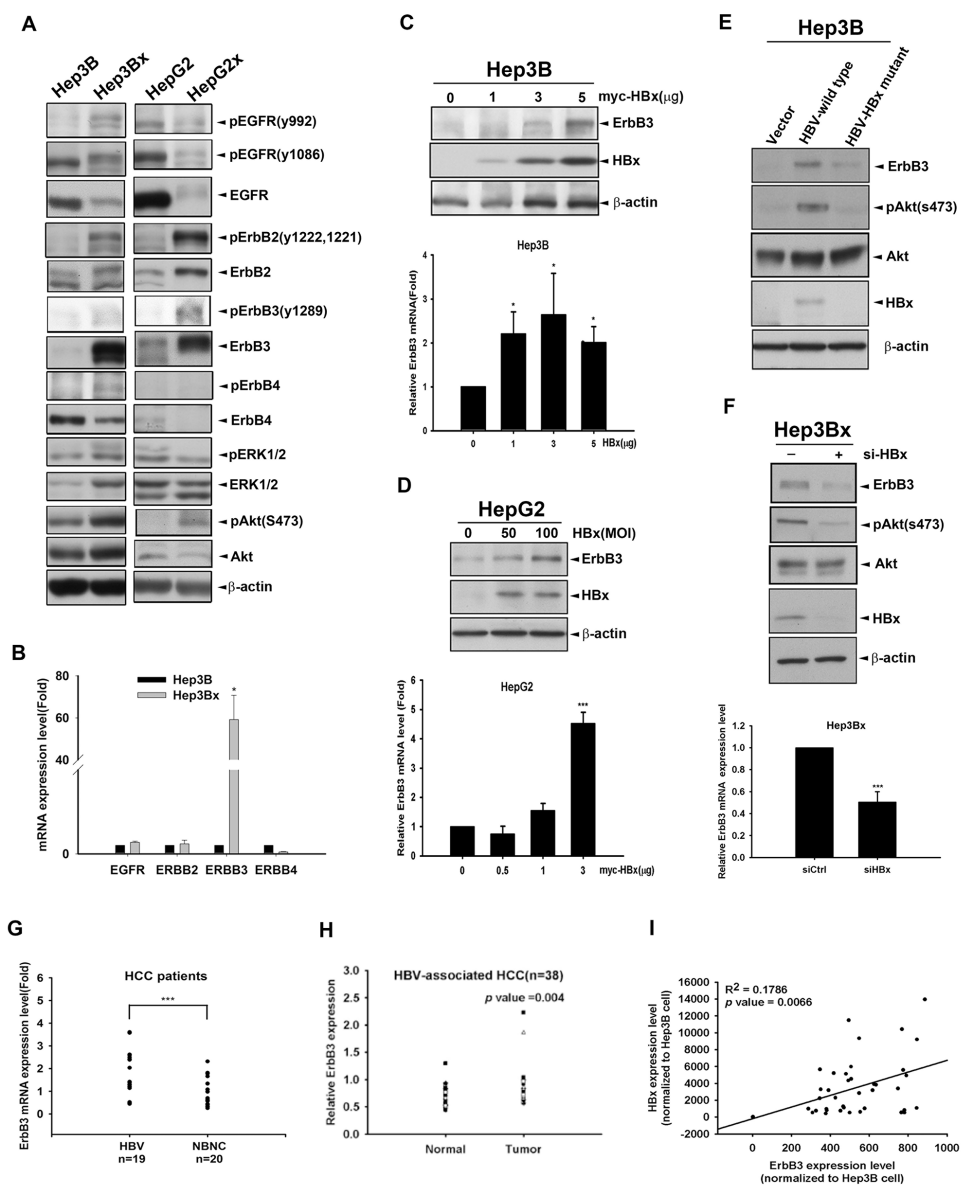
HBx up-regulates ErbB3 expression at both mRNA and protein levels **A.** The expression of ErbB family and downstream signaling in Hep3B and HepG2 cells and their HBx-derivatives were determined by Western blot. **B.** The mRNA expression of ErbB family in Hep3B and Hep3Bx cells were examined by RT-qPCR analysis. The mRNA levels of ErbB family were normalized to GAPDH expression. **C–D.** Hep3B and HepG2 cells were transiently transfected or infected with HBx at different dose for 2 days. The protein expression and mRNA level of ErbB3 were detected by Western blot and RT-qPCR analysis, respectively. **E–F.** Hep3B cells were transfected with control vector, HBV-whole genome, and HBx-deleted HBV mutant for 2 days (E) Hep3Bx cells were transiently transfected with si-HBx for 3 days (F) The protein expression and mRNA level of ErbB3 were detected by Western blot and RT-qPCR analysis, respectively. **G.** The ErbB3 mRNA level of HCC patients were examined by RT-qPCR. The fold changes of ErbB3 mRNA were normalized to its adjacent normal tissue. **H.** The protein expression level of ErbB3 examined by Western blot and quantified by image J system. The expression level of ErbB3 was normalized to β-actin. **I.** Total lysates from HBV-associated HCC tumor liver tissue were prepared and subjected to Western blot with anti-ErbB3, HBx and ERK antibodies. The coefficient of determination (R^2^) between ErbB3 and HBx expression level was analyzed by simple regression, *p* < 0.001.

Since lapatinib is an EGFR/ErbB2 dual inhibitor, we next addressed whether HBx regulates ErbB family expression to sensitize HCC cells to lapatinib. By comparing the ErbB family protein expression and phosphorylation in HBx-overexpressing Hep3Bx and HepG2x and that in their parental cells, our data indicated that HBx decreased EGFR total protein expression but slightly increased phospho-EGFR Tyr992 phosphorylation in Hep3Bx but not in HepG2x cells. In contrast to the inconsistent effect on EGFR phosphorylation, HBx can increase ErbB2 and ErbB3 protein expressions as well as their tyrosine phosphorylation in both Hep3Bx and HepG2x cells. The ErbB2/ErbB3 downstream Akt signaling is also higher in Hep3Bx and HepG2x cells than in their parental cells (Figure 3A). We also analyzed the mRNA level of ErbB family in Hep3B and Hep3Bx cells by using RT-qPCR analysis. We found that mRNA expression of ErbB3 was dramatically higher in Hep3Bx cells than in Hep3B cells (Figure 3B).

Both HBx and ErbB3 have been reported to be capable of inducing Akt activation [44, 45]. The expression of ErbB3 was directly correlated with the degree of sensitivity of breast cancer cell lines to lapatinib [46]. Hence, we suggested that HBx might contribute to lapatinib sensitization through up-regulation of ErbB3 and the subsequent activation of ErbB2/Akt axis. To this end, we first transiently transfected Hep3B and HepG2 cells with HBx followed by Western blot and RT-qPCR analyses. As shown in Figure 3C and 3D, ErbB3 protein and mRNA expression were both induced by HBx in Hep3B and HepG2 cells. Consistently, ErbB3 protein level in Hep3B cells was also induced by HBV whole genome, and this induction was abolished when HBx was mutated (Figure 3E). Moreover, silencing of HBx by siRNA decreased the expression of ErbB3 in both mRNA and protein in Hep3Bx cells (Figure 3F). We then examined the correlation between ErbB3 induction and HBV infection in clinical HCC specimens. The mRNA level of ErbB3 is higher in HBV-associated HCC group in comparison to that in non-virus infection HCC (NBNC) group (Figure 3G). ErbB3 protein expression was also higher in HBV-associated tumor tissues than in their adjacent normal tissues (Figure 3H). In addition, positive correlation between HBx and ErbB3 protein expressions in these HBV-associated tumor tissues was also obtained (Figure 3I). Taken together, these results indicated that ErbB3 is specifically up-regulated by HBx in HCC cells.

### ErbB3 overexpression enhances the sensitization of HCC cell lines to lapatinib

**Figure 4:**
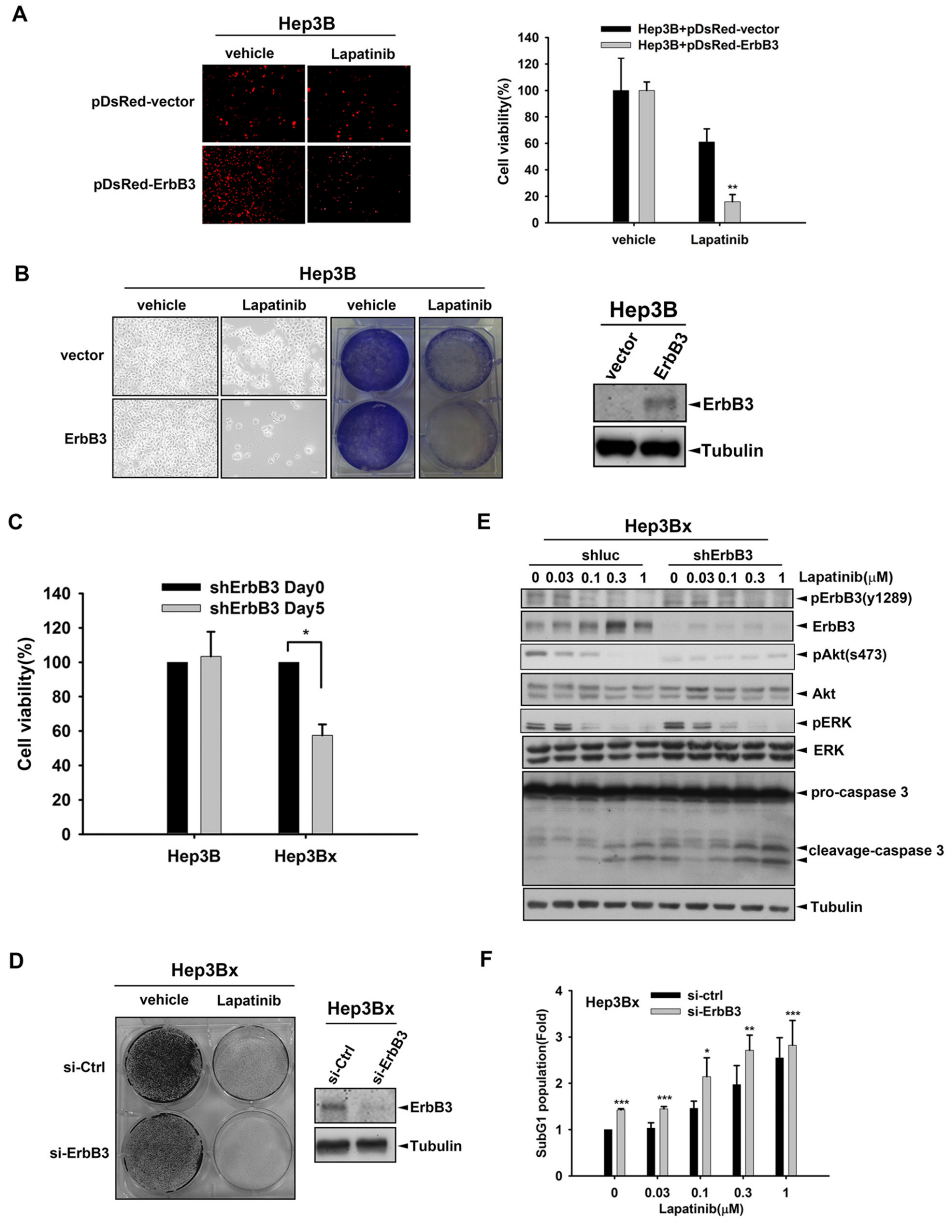
Overexpression of ErbB3 sensitized Hep3B cells to lapatinib **A.** Hep3B cells were transiently transfected with pDsRed vector or pDsred-conjugated ErbB3 for 24 hours followed by 1 μM lapatinib treatment for 5 days. The red fluorescence was detected by fluorescence microscope. **B.** Hep3B cells were transiently transfected with vector control or ErbB3 followed by lapatinib treatment. The cell viability was determined by crystal violet staining. The protein expression of ErbB3 and internal control-Tubulin were detected by Western blot. **C.** Hep3B and Hep3Bx were infected with shErbB3 for 5 days. The cell viability was determined by MTT assay. **D.** Hep3Bx cells were transfected with control or ErbB3 siRNA for 5 days followed by lapatinib treatment. The cell survival was detected by crystal violet staining. The protein expression of ErbB3 and internal control-Tubulin were determined by Western blot. **E.** Hep3Bx cells were infected with luc shRNA and ErbB3 shRNA for 5 days followed by treatment with lapatinib for 24 hours. The protein expression was detected by Western blot. **F.** Hep3Bx cells were transfected with control or ErbB3 siRNA followed by lapatinib treatment for 5 days. The sub-G1 population was determined by PI staining in flow cytometry analysis. Statistical analysis was performed by Student's *t* test. *, *p* < 0.05; **, *p* < 0.01; ***, *p* < 0.001 as compared to control group.

Next, we addressed whether the increased ErbB3 expression is sufficient for the sensitization of HCC cells to lapatinib. As shown in Figure 4A, viability of Hep3B cells expressing DsRed-ErbB3 fusion protein but not DsRed alone were suppressed by lapatinib. In clonogenic assays, the inhibitory effect of lapatinib on the viability of Hep3B cell was also observed obviously after transfection with ErbB3 (Figure 4B). Moreover, silence of ErbB3 by shRNA not only reduced the viability of Hep3Bx cells in MTT assay (Figure 4C), but also synergized the inhibitory effect of lapatinib in clonogenic assays (Figure 4D). However, ErbB3 shRNA did not affect the survival of Hep3B cells (Figure 4C). These findings suggest that HBx renders HCC cells addicted to ErbB3 signaling. In supporting to this finding, silencing of ErbB3 reduced Akt activity and enhanced the lapatinib-induced Caspase3 cleavage in Hep3Bx cells (Figure 4E), indicating the dominance of the pro-survival and anti-apoptotic signaling by HBx-induced ErbB3 in HCC cells. Indeed, lapatinib-induced sub-G1 population was further enhanced by silence of ErbB3 with siRNA in Hep3Bx cells (Figure 4F). Taken together, our data indicated that HBx sensitizes HCC cells to lapatinib through up-regulation of ErbB3 expression.

### Overexpression of HBx enhanced the dimerization of ErbB2 and ErbB3

**Figure 5:**
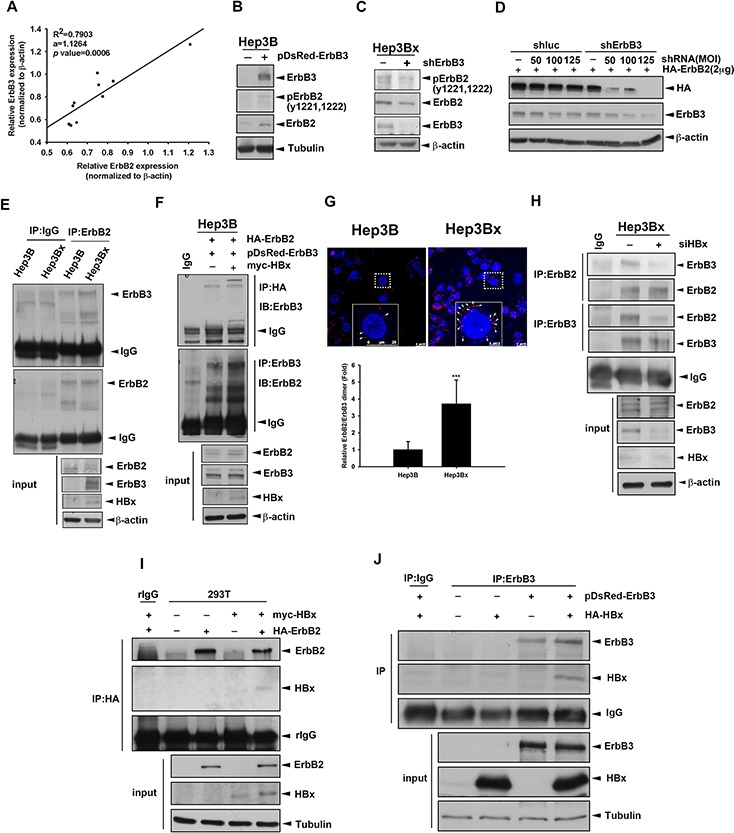
Overexpression of HBx enhanced the dimerization of ErbB2 and ErbB3 **A.** The protein expression in human HCC cell lines was examined by Western blot and quantified by image J system. The expression level of ErbB2 and ErbB3 were normalized to its β-actin. The coefficient of determination (R^2^) between ErbB3 and ErbB2 expression level was analyzed by simple regression, *p* < 0.001. **B.** Hep3B cells were transfected with DsRed vector or DsRed fused ErbB3. The protein expression was determined by Western blot. **C.** Hep3Bx cells were infected with shRNA, the protein expression was determined by Western blot. **D.** Hep3Bx cells were infected with shRNA and transfected with HA-ErbB2 for 2days. The protein expression was determined by Western blot. **E.** Total lysate prepared from Hep3B and Hep3Bx cells were subjected to IP/Western blot analysis. **F, I.** and **J.** Hep3B (F) or HEK293T (I and J) cells were transient transfected with ErbB2, ErbB3, and vector or HBx, respectively. The total lysates were harvested for IP/Western blot with indicated antibodies. **G.** Hep3B and Hep3Bx were seeded for 24 hour and detected the interaction of ErbB3 and ErbB2 by using PLA kit. The red fluorescence was detected by confocal microscope. **H.** Hep3Bx cells were transfected with control or ErbB3 siRNA for 3 days. The total lysates were harvested for IP/Western blot with indicated antibodies.

ErbB3 does not possess tyrosine kinase activity and drives downstream signaling through heteromeric association with other ErbB family members [47–49]. Since ErbB3 expression is up-regulated in accompany with the kinase activation of ErbB2 but not EGFR and ErbB4 in response to HBx overexpression (Figure 3A), hence we further investigated whether HBx promotes the up-regulated ErbB3 to form a activated complex with ErbB2. First, we examined the correlation between ErbB2 and ErbB3 in various HCC cell lines, and positive correlation between ErbB3 and ErbB2 protein levels was found (Figure 5A). Ectopic expression of ErbB3 in Hep3B cell also increased the protein and tyrosine kinase activity of ErbB2 (Figure 5B). On the contrary, silencing ErbB3 by shRNA reduced ErbB2 protein and tyrosine kinase activity expression (Figure 5C). In addition, silence of ErbB3 by shRNA specifically targeting its 3′UTR also decreased ectopic expression of ErbB2, indicating that ErbB3 affects the stability of ErbB2 at post-transcriptional level (Figure 5D).

ErbB3 prefers to form heteromeric protein complex with ErbB2 [48]. Here, we further investigate whether overexpression of HBx enhanced the interaction between ErbB2 and ErbB3 in IP/WB analysis, and the results showed that the co-immunoprecipitated ErbB3 in the anti-ErbB2 immunocomplex was more abundant in Hep3Bx cells than in Hep3B cells (Figure 5E). Furthermore, overexpression of HBx can increase the interaction between ectopically expressed ErbB2 and ErbB3 protein in the reciprocal IP/WB analysis (Figure 5F). In proximity ligation assay (PLA), the ErbB2/ErbB3 protein interaction (red spot) was not only located on the plasma membrane but also was more abundant in Hep3Bx cells than in Hep3B cells (Figure 5G). In contrast, silence of HBx by siRNA significantly decreased the interaction between these two receptors in Hep3Bx cells (Figure 5H). We further examined whether HBx physically interacts with ErbB2 and ErbB3 by co-transfection of HBx with ErbB2 or ErbB3 into HEK293T cell, and found that HBx can be detected in the anti-ErbB2 and anti-ErbB3 immunoprecipitates (Figure 5I and 5J). These results indicated that HBx protein enhances the ErbB2/ErbB3 complex formation by physically protein-protein interaction with both of them.

### HBx induced ErbB3 expression through enhancing ErbB3 promoter activity

**Figure 6:**
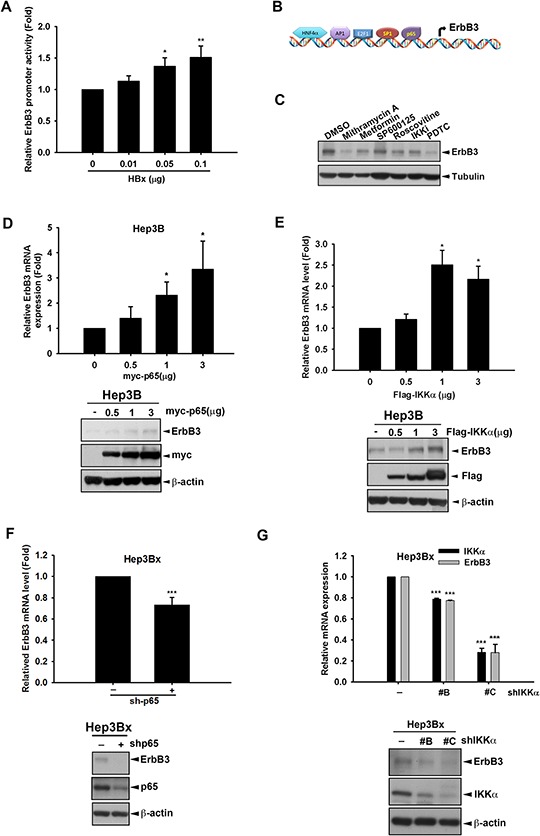
α/NF-κB HBx mediates HBx-induced ErbB3 transcription **A.** HEK293T cells were transfected with indicated concentration of HBx, promoter control or *ErbB3* promoter-luciferase, respectively. The culture medium was prepared for detecting promoter activity and the total lysates were harvested for renilla activity. **B.** The putative transcription factor for *ErbB3* promoter was illustrated. **C.** Hep3Bx cells were treated with Mithramycin A 0.3 μM, Metformin 5 mM, SP600125 30 μM, Roscovitine 20 μM, IKKi 10 μM, and PDTC 10 μM for 24 hr. **D–G.** Hep3B cells were transfected with myc-p65 or Sp-1 in different dose for 48 hr (D) Hep3B cells were transfected with indicated dose of Flag-IKKα for 48 hr (E) Hep3Bx cells were infected with p65 shRNA for 3 days (F) Hep3Bx cells were infected with three different clones of IKKα shRNA for 3 days (G) The ErbB3 mRNA and protein expression level were examined by RT-qPCR and Western blot. Statistical analysis was performed by Student's *t* test. *, *p* < 0.05; **, *p* < 0.01; ***, *p* < 0.001 as compared to control group.

Next, we investigated whether HBx transcriptionally up-regulates ErbB3 expression through enhancing its promoter activity. HEK293T cells were co-transfected with HBx and the luciferase construct containing the essential regulatory region of *ErbB3* promoter [50]. As shown in Figure 6A, the *ErbB3* promoter activity was increased in HBx-expressing cells in a dose-dependent manner, indicating that HBx transcriptionally increased ErbB3 gene expression. HBx has been reported to activate various transcription factors such as AP-1, NF-κB, and CREB [51–53]. By using JASPAR database, the putative transcription factors binding to *ErbB3* promoter were predicted (Figure 6B), and among them Sp-1, NF-κB, AP1, E2F1, and HNF4α have been reported to be activated in response to HBx expression [54–58]. After screening with the inhibitors of these transcription factors, ErbB3 expression was dramatically decreased by pyrrolidine dithiocarbamate (PDTC) and mithramycin A, specific inhibitors against NF-κB and Sp1 respectively (Figure 6C). Overexpression of p65 but not Sp-1 enhanced both ErbB3 mRNA and protein levels (Figure 6D and data not shown). In addition to activating NF-κB through phosphorylation of IκB in the cytoplasm [59], IKKα has been reported to function as a epigenetic regulator for NF-κB-dependent gene transcription in the nucleus in response to HBx overexpression [42, 60]. Our result showed that overexpression of IKKα can induce ErbB3 expression at both mRNA and protein levels in a dose-dependent manner (Figure 6E). In contrast, silencing of p65 by shRNA reduced both ErbB3 mRNA and protein expressions (Figure 6F). Moreover, silence of IKKα also suppressed ErbB3 protein level in Hep3Bx cells (Figure 6G). It has been reported that IKKβ, another subunit of IKK complex, activates p65 activity through phosphorylation of IκBα. Here we also determined whether IKKβ also involved in ErbB3 up-regulation. Silence of IKKβ by using shRNA obviously suppressed ErbB3 expression (Supplementary Figure S2B). On the contrary, overexpression of IKKβ increased ErbB3 expression (Supplementary Figure S2C). These data suggest that IKK/NF-κB is involved in HBx-mediated ErbB3 expression.

**Figure 7:**
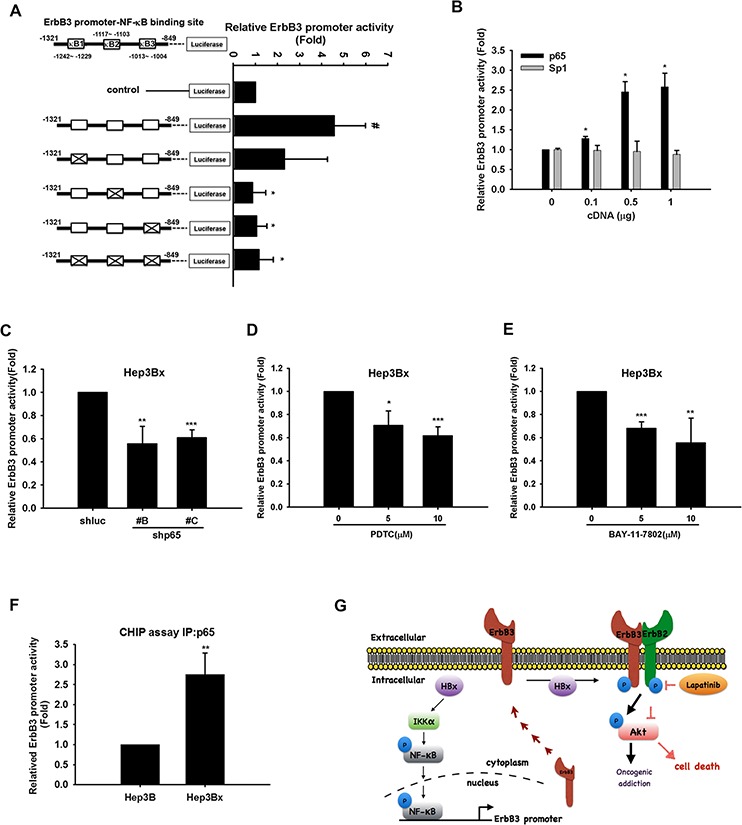
NF-κB directly binds to and up-regulates *ErbB3* promoter **A–E.** HEK293T cells were transfected with, control or *ErbB3* promoter-luciferase with or without mutations at putative κB binding sites 1, 2, and 3 (A) HEK293T cells were transfected with control or *ErbB3* promoter-luciferase and indicated doses of myc-p65 or Sp-1 (B) Hep3Bx cells were transduced with p65 shRNA (C) or were treated with PDTC (D) or BAY-11–7802 (E) for 48 hr after transfection with *ErbB3* promoter-luciferase. The culture medium was prepared for detecting promoter activity and the total lysates were harvested for renilla activity. **F.** Total lysate of Hep3B and Hep3Bx were harvested for chromatin IP analysis with anti-p65 antibody, and the binding activity of p65 on *ErBb3* promoter was determined by qPCR analysis. Statistical analysis was performed by Student's *t* test. *, *p* < 0.05; **, *p* < 0.01 as compared to control group. **G.** Diagrammatic illustration of the hypothetical model derived from this study.

By using JASPAR database, there are three predicted p65-binding sites (κB1: −1242 to −1229; κB2 −1117 to −1103; κB3 −1013 to −1004) on *ErbB3* promoter. Our results found that mutation of κB2 or κB3 but not κB1 significantly decreased *ErbB3* promoter activity (Figure 7A). Overexpression of p65 but not Sp1 enhanced *ErbB3* promoter activity in a dose-dependent manner in HEK293T cells (Figure 7B). In contrast, silence of p65 by two different clones of shRNA decreased *ErbB3* promoter activity in Hep3Bx cells (Figure 7C). Furthermore, treatment with PDTC and BAY-11–7802 to respectively inhibit NF-κB and IKKα activation in Hep3Bx cells also suppressed ErbB3 promoter activity (Figure 7D and 7E). To further demonstrate that ErbB3 expression was transcriptionally regulated by NF-κB *in vivo*, the binding activity of p65 on *ErbB3* promoter was determined in ChIP assays, and the results showed that the association of p65 to the *ErbB3* promoter was obviously higher in HBx-overexpressing Hep3Bx cells than in Hep3B cells (Figure 7F). These data suggest that NF-κB directly binds to *ErbB3* promoter and regulates ErbB3 expression in response to HBx expression.

## DISCUSSION

EGFR inhibitors such as erlotinib, lapatinib, and cetuximab alone or in combination with sorafenib have been tested for HCC treatment [61], but did not provide additional benefit to these patients [62]. EGFR mutation or ErbB2 overexpression has been viewed as critical biomarkers for treatment of EGFR TKIs in NSCLC and breast cancer patients [34]. However, such mutations were not found in HBx-expressing HCC cells, and also were not associated with the therapeutic efficacy of EGFR TKIs in HCC patients [63, 64]. Our data unexpectedly found that HBx can sensitize HCC cell lines to EGFR/ErbB2 TKIs, including lapatinib, erlotinib, and gefitinib, and suggest ErbB3 overexpression as a potential biomarker to predict the therapeutic efficacy of lapatinib in HCC patients.

Our data showed that HBx-expressing HCC cells were oncogenically addicted to ErbB3/ErbB2 signaling pathway as evidenced by the findings that silence of ErbB3 and treatment with lapatinib synergistically induced cell death in HBx-expressing cells. Like ErbB3 induction by HBx, ectopic overexpression of ErbB3 also increased the oncogenic addiction of HCC cells to this RTK, and thereby sensitizes HCC cells to lapatinib. Mechanistically, HBx increased ErbB3 protein expression and mRNA level through enhancing its promoter activity, and also augmented the formation of ErbB2/ErbB3 complex through protein-protein interactions with these receptors.

In this study, we identified NF-κB as the key transcription factor for *ErbB3* promoter activation. Although our data also showed that mithramycin A inhibited both Sp1 and ErbB3 protein expression (Figure 6C), *ErbB3* promoter activity was not increased by overexpression of Sp1 (Figure 7B). Moreover, mithramycin A has been reported to suppress Sp1 activity as well as TNF-α-induced NF-κB activation [65]. Our data also showed that mithramycin A reduced p65 activity in Hep3Bx cells (Supplementary Figure S2A). These observations suggest that NF-κB, but not Sp1, plays a critical role in mediating HBx-induced ErbB3 expression. In addition to inducing ErbB3 gene transcription, HBx also increased the protein expression of DsRed-ErbB3 (Supplementary Figure S3A). Moreover, silencing of HBx in Hep3Bx cells decreased ErbB3 protein stability in the presence of cycloheximide (Supplementary Figure S3B), suggesting the existence of post-translational modification. Our data further demonstrate that HBx can physically bind to both ErbB3 and ErbB2 to enhance the ErbB3/ErbB2 interaction (Figure 5I and 5J). These results revealed that HBx dually up-regulates ErbB3 at both transcriptional and posttranslational levels, and explain why the augmenting effect of HBx on ErbB3 promoter activity is not parallel to that on ErbB3 protein expression.

Although HBx-expressing cells are more sensitive to lapatinib, our data also showed that erlotinib and gefitinib did not completely suppress the viability of cells even at high dose. In contrast to the up-regulation of ErbB2 and ErbB3, our data showed that EGFR expression level is decreased by HBx (Figure 3A) probably due to the suppression by HBx-induced miR-7 [42], suggesting possible reason for the incompletely suppressive effect of erlotinib and gefitinib on viability of HBx-expressing cells at high dose.

Several potential reasons could explain why the therapeutic benefits of EGFR/ErbB2 TKIs on HCC patients were not observed in clinical trials. Recruitment of non-HBV-associated HCC patients in the clinical trials would be the first possible reason for the inefficacy of EGFR TKIs [62]. Another plausible reason is that the HBV DNA level or HBsAg expression, which is practically measured in clinic, may not be actually associated with the HBx expression in HCC tissues. Expression of HBx is cyclic but not steady when HBV is replicated in HCC cells [66]. It has also been found that 32% of HBV-associated HCC patients express HBx with C-terminal deletion [67], which reduces the transactivation activity of HBx [68, 69]. Therefore, the dynamic or truncated expression of HBx in HCC tissues of HBV-positive patients may not be able to induce ErbB3 expression and sensitize HCC cells to lapatinib. These findings indicate the limitation of HBx as the biomarker to predict the therapeutic efficacy of lapatinib in HCC patients. Although our data showed clinical correlation between HBx and ErbB3 expressions, this finding should be further validated due to the limited number of human HCC samples used in this study.

In summary, our study indicated that HBx increases ErbB3 expression through IKKα/NF-κB signaling pathway and enhances the formation ErbB2/ErbB3 complex by physically protein-protein interaction. This effect thereby shifts the oncogenic addiction of HCC cells to ErbB2/ErbB3 and sensitizes them to lapatinib (Figure 7E). Furthermore, HBx and ErbB3 overexpression could serve as potential biomarkers for predicting therapeutic efficacy of EGFR/ErbB2 inhibitors in HCC patients.

## MATERIALS AND METHODS

### Cell lines and cell culture

Human hepatocellular carcinoma cell lines, Hep3B, HepG2, their derivatives with HBx expression were cultured in Dulbecco's Modified Eagle Medium: Nutrient Mixture F-12 (DMEM/F12) and supplemented with 10% fetal bovine serum (Gibco), 100U/ml penicillin, and 100U/ml streptomycin. SK-Hep1, Tong, Mahlavu, HCC36 were cultured in Dulbecco's Modified Eagle Medium (DMEM) and supplemented with 10% fetal bovine serum (Gibco), 100U/ml penicillin, and 100U/ml streptomycin.

Huh7 was cultured in Dulbecco's Modified Eagle Medium (DMEM) with 1% L-glutamine and supplemented with 10% fetal bovine serum (Gibco), 100U/ml penicillin, and 100U/ml streptomycin. HA22T/VGH was cultured in Dulbecco's Modified Eagle Medium (DMEM) with 1% L-glutamine, 1% Non-Essential Amino Acid and supplemented with 10% fetal bovine serum (Gibco), 100U/ml penicillin, and 100U/ml streptomycin. These cells are all incubated at 37°C in a humidified atmosphere of 95% air and 5% CO_2_.

### Transfection

Cells were transfected with plasmid or si-RNA by using Lipo-Fectamine 2000 (invitrogen) or *Trans*IT-X2 (Mirus) transfection reagent according to the manufacturer's instruction. After 48 hr of transfection, cells lysates were harvested and subjected to analysis. Oligonucleotide used for PCR, mutagenesis, or RNA interference were listed in Supplementary Tables S1–S3.

### Preparation of cell lystes

The cell lysates were extracted in RIPA buffer (50 mM pH7.4 Tris-HCl, 1% Nonidet P-40, 0.15% Na-DOC, 150 mM NaCl and 1 mM EDTA) with protease inhibitors, homogenized with sonication and store at −30°C.

### Western blot and antibody

The concentration of protein was measured by Bradford protein assay (Bio-Rad protein assay). Protein was denatured in 1X sample buffer at 95°C for 5 minutes, and separated by SDS-PAGE followed by transfer to a PVDF membrane (0.45 μm, millipore) or NC membrane (0.22 μm, GE Healthcare). The membrane was blocked with 5% milk in Tris-Bufferd Saline-Tween (TBST; 10mL of 2 μM Tris-HCL pH7.4, 100mL of 5M NaCl, 0.5mL of 100% Tween-20, and 890mL ddH_2_O) for an hour at room temperature. After blocking, the membrane was immunostained at 4°C overnight with the indicated antibodies in a dilution from 1:1000 to 1:5000. After incubation with HRP labeled secondary antibody for an hour, the chemiluminscence signal was catalyzed by ECL (GE Healthcare or Millipore), and detected the level of protein expression. Phospho-EGFR Tyr992 (#2235), Phospho-EGFR Tyr1068 (#2236), Phospho-EGFR Tyr1086 (#2220), Phospho-ERBB2 Tyr1221/1222 (#2243), Phospho-ERBB3 Tyr1289 (#4791), Akt (#9272), Phospho-Akt ser473 (XP) (#9271), Erk (#9102), and Phospho-Erk T202, Y204 (#9106s), and PARP (#9542) were purchased from Cell signaling Technology. EGFR (sc-03), ERBB2 (sc-284), ERBB3 (sc-285) were purchased from Santa Cruz Biotechnology. Hepatitis B virus X (ab39716) was purchased from Abcam. α-Tubulin (T6074) and β-actin (A2228) were purchased from SIGMA. Caspase-3 IMG-144A was purchased from IMGENEX. Anti-c-myc (Cat No. 11667203001) was purchased from Roche.

### MTT assay

Cells were seeded at density of 3 × 10^3^~1 × 10^4^ cells/well in 96-well plate. To identify the different viability between the parental cells and HBx-expressing cells after the treatment, the culture medium was removed and 80 μl of serum-free medium and 20 μl of 5 mg/mL MTT solution (Sigma) were mixed and added to each well followed by incubation in 37°C for 3 hours. Then, MTT solution was removed and 100 μl of DMSO was added to lyse the cells. After incubation for one hour, the absorbance were be detected by ELISA reader.

### Cell counting analysis

The cultured medium was removed and cells were washed with phosphate buffer saline (PBS) followed by addition of appropriate trypsin-EDTA to de-attach cells from dishes. Next, cells were collected in 1.5mL eppendorf and centrifuged at 16,000g for 5 minutes. Supernatant were removed and the cell pallet was scattered in 100 μl fresh medium, and cells were diluted with trypan blue with ratio 1:1. Finally, cells were count by Countess^TM^ automated cell counter (Invitrogen, Carlsbad, CA).

### Cell cycle analysis

Cells were treated with lapatinib in different dosages for 5 days. After treatment, cells were washed with PBS and trypsinized. Then, cells were collected and centrifuged followed by supernatant removal, and then 75% ethanol was added to fully fix cells. Before analyzed by flow cytometry, the cells were stained with PI buffer (1% Triton-X 100, 0.1%mg/mL PureLink^TM^ RNase A (Cat no. 12091-021), and 20 μg/mL Propidium iodide in 1X PBS) for 30 minutes, and analyzed by Flow cytometry.

### Cell apoptosis assay

Hep3B and Hep3Bx cells were treated with lapatinib for 5 days followed by wash with PBS and trypsinization. Cells were collected and cell viability was measured by staining with Annexin V-FITC Apoptosis Detection Kit (BioVision, Cat#K101-400) for 15 min at room temperature.

### RNA extraction

Cells were washed with PBS and added 0.5 mL Trizol^TM^ reagent (Roche) to extract total RNA followed by mix with 0.1 mL of chloroform to separate aqueous phase, interphase, and organic phase. Next, total RNA was precipitated from the aqueous phase by mixing with 0.25 mL of isopropanol. The pellet was washed with 0.5 mL 75% EtOH for twice followed by air-dried and dissolved in DEPC-treated water.

### RT-qPCR (reverse transcription-quantitative polymerase chain reaction)

After total RNA extraction, reverse transcription polymerase chain reaction was performed. The reagents in RT-PCR analysis include 1 μg sample, DEPC H_2_O, Oligo-dT, dNTP, DTT, 5X MMLV buffer, and MMLV transcriptase. All components were purchased from Roche. The cDNA products were used for real-time PCR. All sample were 10X dilute from real-time PCR product. For quantitative real-time polymerase chain reaction, SYBR Green Master Mix was used to detect the expression of indicated genes with specific primers described in the supplementary information.

### HCC patient samples

The patient samples of HCC (39) and HBV-associated HCC (88) were from National Cheng Kung University Hospital and National Health Research Institutes. Informed consents were signed by patients with approval by the Institutional Review Board, China Medical University Hospital, Taichung, Taiwan (DMR101-IRB1-119) and by the Institutional Review Board of the Human Investigation Committee of College of Medicine, National Cheng Kung University Tainan, Taiwan (B-ER-102-210). The expressions of ErbB3 mRNA normalized respectively to GAPDH were determined in RT-qPCR analysis. The protein expression of ErbB3 and HBx were analyzed by Western blot and quantified by image J analysis. The differences in various HCC tumor tissues compared to the normal tissues was determined by a two-tailed Student's t test and *p* < 0.001 was defined as statistically significant.

### Chromatin immuno-precipitation (ChIP)

Cells were fixed with 37% formadehyde for 15 minutes at room temperature, and 1M glycine was used to stop the reaction followed by wash with ice-cold PBS twice. Cells were scraped off and washed by IP buffer for 3 times before sonication. Total lysate was added with 1mL ChIP buffer and sonicated at 30 Amp for total 3 minutes. After quantification, 1mg total lysates were immunopreciated with indicated antibody or IgG at 4°C overnight. The immunoprecipitated DNA was extracted and subjected to quantitative PCR with indicated primers.

### Luciferase assay

Cells were seeded at density of 3 × 10^4^ cells/well in 12-well plate. ErbB3 promoter luciferase DNA and HBx DNA were co-transfected into cells for 48 hours. The luciferase activity was measured by using Secrete-Pair^TM^ Gaussia Luciferase Assay Kit (GeneCopoeia^TM^) according to the manufacturer's protocol. For luciferase reporter assay, firefly luciferase activities were normalized with the renilla activities.

### Crystal violet staining assay

To access the survival and sensitivity of cells to EGFR inhibitors, cells were seeded at low density and treated with lapatinib for 5 days. Crystal violet was used to stain live cells. The quantitation was performed by solving crystal violet with 30% acetic acid and measured by ELISA reader.

### Proximity ligation assay (PLA)

Cells were seeded at density of 3 × 10^4^ cells/slide. Fixed with 4% paraformaldehyde for 10 minutes at room temperature and blocking with blocking solution (Duolink^®^ *In Situ*, Sigma) for 30 minutes at 37°C on the next day. The slides were immunostained at 4°C overnight with anti-ErbB3 and anti-ErbB2 specific antibodies in a dilution of 1:100. Added PLA probe solution (Duolink^®^ *In Situ*, Sigma) and then ligation-ligase solution (Duolink^®^ *In Situ*, Sigma) for 1 hour and 30 minutes, respectively. Amplify the signal by incubated with amplification-polymerase solution (Duolink^®^ *In Situ*, Sigma) at 37°C for 100 minutes. The visual spot were determined by confocal microcopy at absorbance 624 nm.

### Statistical analysis

The difference in relative gene expression between tumor and normal tissues was calculated by a two-tailed Student's t test. Coefficient analyses were performed for the correlation between gene expressions. All these statistical analyses were performed using Sigma Plot 10.0. *p* < 0.05 was defined as statistically significant.

## SUPPLEMENTARY FIGURES AND TABLES




