# Supplementary material for: HBx sensitizes hepatocellular carcinoma cells to lapatinib by up-regulating ErbB3
Source: Oncotarget. 2015 Nov 16;7(1):473–89. doi: 10.18632/oncotarget.6337 (PMC4808012; doi:10.18632/oncotarget.6337)
Supplement: Supplementary file 1 [file oncotarget-07-0473-s001.pdf]

SUPPLEMENTARY FIGURES AND TABLES

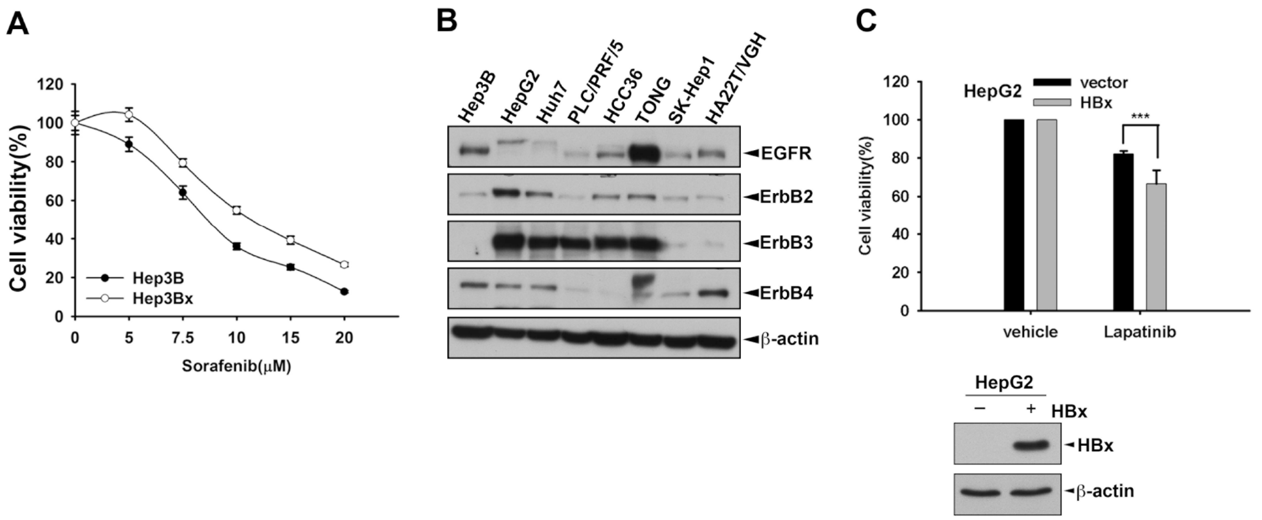

Supplementary Figure S1: Effects of HBx on the sensitivity of HCC cell lines to RTK TKIs. A. The cell viability of Hep3B and Hep3Bx cells was detected by MTT assay after treatment with sorafenib for 3 days. **B.** Total lysate prepared from HCC cell lines was subjected to western blot analysis with anti-ErbB family and anti-Δ-actin antibodies. **C.** HepG2 cells were infected by HBx-expressing lentivirus and treatment with lapatinib for 3 days. Cell viability was determined by MTT assay and the protein expression of HBx and Δ-actin were examined by western blot.

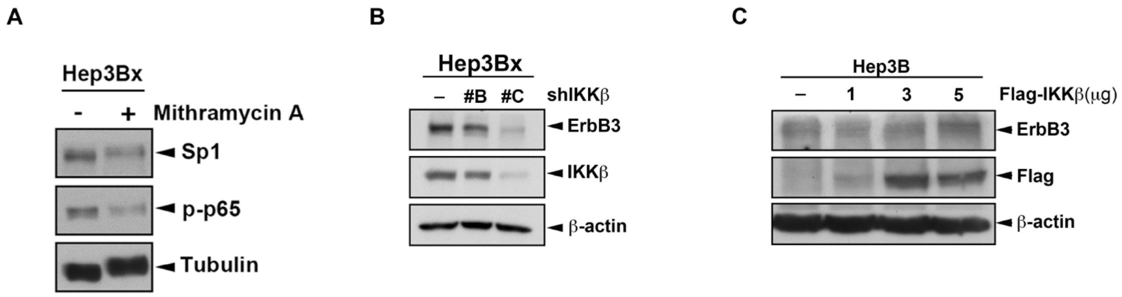

Supplementary Figure S2: Mithramycin inhibits both Sp1 expression and NF-κB activity. A. Hep3Bx cells were treated with Sp1 inhibitor-Mithramycin A for 24 hour. Total lysate was prepared and subjected to Western blot with anti-Sp1, anti-p-p65 and anti-tubulin antibodies. B-C, Hep3Bx cells were infected with three different clones of IKKΔ shRNA for 3 days **B.** Hep3B cells were transfected with Flag-IKKΔ for 2 days **C.** The ErbB3 protein expression level was examined by Western blot.

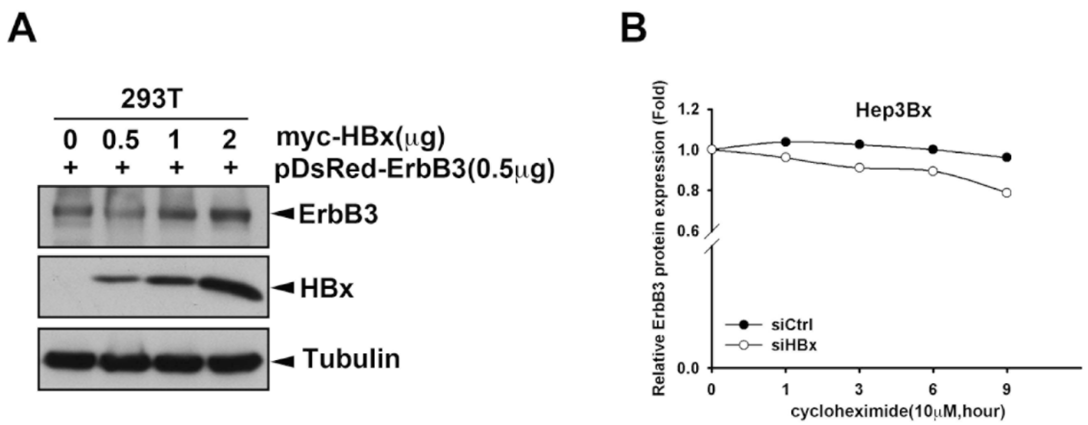

Supplementary Figure S3: HBx increased ErbB3 expression on post-transcriptional level. **A.** HEK293T cells were transfected with pDsRed-ErbB3 and different dose of myc-HBx. Total lysate was prepared and subjected to Western blot with anti-ErbB3, anti-HBx and anti-tubulin antibodies. **B.** Hep3Bx cells were transfected with control or HBx siRNA followed by treatment with 10μM cycloheximide. Protein expression of ErbB3 was analyzed by western blot and quantified by Image J system.

Supplementary Table S1: Specific primers used in qPCR of this study.

Supplementary Table S2: Specific primers used for mutagenesis of NF-κB binding site on *ErbB3* promoter.

Supplementary Table S3: The oligonucleotide sequence of siRNA or shRNAs used in this study.
