# Supplementary material for: HBx sensitizes hepatocellular carcinoma cells to lapatinib by up-regulating ErbB3
Source: Oncotarget. 2015 Nov 16;7(1):473–89. doi: 10.18632/oncotarget.6337 (PMC4808012; doi:10.18632/oncotarget.6337)
Supplement: Supplementary file 2 [file oncotarget-07-0473-s002.docx]

**Supplementary Table S1 Specific primers used in qPCR of this study**

|  |  | Sequence (5’-3’) |
| --- | --- | --- |
| EGFR | F | CTCCTCTTGCTGCTGGTGGT |
|  | R | AAGAGAGCTTGGTTGGGAGC |
| ErbB2 | F | GACCTGCTGAACTGGTGTAT |
|  | R | ACTCTGTCTCGTCAATGTCC |
| ErbB3 | F | CCAAGACCATCTGTGCTCCT |
|  | R | TTGTCAGGAGGACAGGCCCT |
| ErbB4 | F | AGCCCGTAATGTCTTAGTGA |
|  | R | GATGGGTGAATTTCCTGTAA |
| IKKα | F | ACCATTTGCATCCAGAAGTTTTATC |
|  | R | TGCTCAGGTGACCAAACAGCT |
| p65 | F | CTGCAGTTTGATGATGAAGA |
|  | R | TAGGCGAGTTATAGCCTCAG |
| κB site 1 on *ErbB3* promoter | F | ACAACTAGGCTCTCCGGGCGAGAT |
|  | R | TTGCTGCCCAAAGCCCTGCTAG |
| κB site 2 on *ErbB3* promoter | F | CCAGACTCCAGTGTGGAAGG |
|  | R | CGTAGGACATCGAGGCAAGA |
| κB site 3 on *ErbB3* promoter | F | TCTCCGCGTCCCACTTCACT |
|  | R | TTCCACACTGGAGTCTGGCC |
| GAPDH | F | AGCCACATCGCTCAGACAC |
|  | R | GCCCAATACGACCAAATCC |

**Supplementary Table S2. Specific primers used for mutagenesis of NF-κB binding site on *ErbB3* promoter.**

| **κ**B site 1 | F’: 5'-GAAATGCAAGGCCGTCTTTCACTCCACTGCCACGGATG-3' |
| --- | --- |
|  | R’: 5'-CATCCGTGGCAGTGGAGTGAAAGACGGCCTTGCATTTC-3' |
| **κ**B site 2 | F’: 5'-CCGGGCAGCTGGTGGTTCAGGTTCCAGAGGTCC-3' |
|  | R’: 5'-GGACCTCTGGAACCTGAACCACCAGCTGCCCGG-3' |
| **κ**B site 3 | F’: 5'-GCGAGAGCCTGGACTTTTAAGGCACCTGGGAGGG-3' |
|  | R’: 5'-CCCTCCCAGGTGCCTTAAAAGTCCAGGCTCTCGC-3' |

**Supplementary Table S3. The oligonucleotide sequence of siRNA or shRNAs used in this study.**

|  | Sequence (5’-3’) |
| --- | --- |
| ErbB3 siRNA | F’-GCGAUGCUGAGAACCAAUA |
|  | R’UAUUGGUUCUCAGCAUCGC |
| siHBx siRNA | F’-CCAAUUUCCAGAAACAUAA |
|  | R’-UUAUGUUUCUGGAAAUUGG |
| IKKα shRNA-B | CCGGCCAGATTATGAAGAAGTTGAACTCGAGTTCAACTTCTTCATAATCTGGTTTTT |
| IKKα shRNA-C | CCGGCCAGCCTCTCAATGTGTTCTACTCGAGTAGAACACATTGAGAGGCTGGTTTTT |
